# Towards an intelligent malaria outbreak warning model based intelligent malaria outbreak warning in the northern part of Benin, West Africa

**DOI:** 10.1186/s12889-024-17847-w

**Published:** 2024-02-13

**Authors:** Gouvidé Jean Gbaguidi, Nikita Topanou, Walter Leal Filho, Guillaume K. Ketoh

**Affiliations:** 1 Department of Geography, West African Science Service Centre On Climate Change and Adapted Land Use (WASCAL), Faculty of Human and Social Sciences, University of Lomé, Lomé, Togo; 2 Department of Zoology, Laboratory of Ecology and Ecotoxicology, Faculty of Sciences, University of Lomé, 1BP: 1515 Lomé, Togo; 3 Department of Chemistry, Kaba Laboratory of Chemical Research and Application (LaKReCA), Faculty of Science and Technic of Natitingou, University of Abomey, Abomey, Benin; 4 Research and Transfer Centre Sustainability and Climate Change Management, Faculty of Life Sciences, Hamburg University of Applied Sciences, Ulmenliet 20, 21033 Hamburg, Germany

**Keywords:** Climate change, Malaria, Prediction, Northern Benin

## Abstract

**Background:**

Malaria is one of the major vector-borne diseases most sensitive to climatic change in West Africa. The prevention and reduction of malaria are very difficult in Benin due to poverty, economic insatiability and the non control of environmental determinants. This study aims to develop an intelligent outbreak malaria early warning model driven by monthly time series climatic variables in the northern part of Benin.

**Methods:**

Climate data from nine rain gauge stations and malaria incidence data from 2009 to 2021 were extracted from the National Meteorological Agency (METEO) and the Ministry of Health of Benin, respectively. Projected relative humidity and temperature were obtained from the coordinated regional downscaling experiment (CORDEX) simulations of the Rossby Centre Regional Atmospheric regional climate model (RCA4).

A structural equation model was employed to determine the effects of climatic variables on malaria incidence. We developed an intelligent malaria early warning model to predict the prevalence of malaria using machine learning by applying three machine learning algorithms, including linear regression (LiR), support vector machine (SVM), and negative binomial regression (NBiR).

**Results:**

Two ecological factors such as factor 1 (related to average mean relative humidity, average maximum relative humidity, and average maximal temperature) and factor 2 (related to average minimal temperature) affect the incidence of malaria. Support vector machine regression is the best-performing algorithm, predicting 82% of malaria incidence in the northern part of Benin.

The projection reveals an increase in malaria incidence under RCP4.5 and RCP8.5 over the studied period.

**Conclusion:**

These results reveal that the northern part of Benin is at high risk of malaria, and specific malaria control programs are urged to reduce the risk of malaria.

## Introduction

The impacts of climate change on human health have attracted more attention in recent years. There is increasing evidence of adverse effects of climate change on health worldwide, both direct effects and indirect effects mediated by disruption in ecological and socioeconomic systems.

The warming of our planet due to the emission of greenhouse gases (GHGs) is now a major challenge for countries worldwide. Due to its low adaptive capacity and high sensitivity of socioeconomic systems, Africa is the most vulnerable continent to the impacts of climate change [1, 2].

Malaria is considered one of the major vector-borne diseases most sensitive to changes in environmental conditions such as climate variables and land use change. The transmission of malaria is linked with changes in temperature, rainfall, humidity and the level of immunity in humans [3, 4]. Socio economic factors also influence the occurrence of malaria in both rural and urban areas [5, 6]. The burden of mortality and morbidity is worse in poor countries, especially in West Africa [5].

Malaria is a vector-borne disease caused by the infection of red blood cells with protozoan parasites of the genus Plasmodium, where the parasites enter the human body through the bite of an infected species of Plasmodium that infects humans. The driver of malaria in Africa is the mosquito of the Anopheles Plasmodium falciparum, which causes most of the severity and deaths attributable to the disease and resists treatment [3]

Despite the efforts made over the decades by the Benin government, malaria caused 95% of deaths. Northern provinces have the highest prevalence of malaria in Benin [7]. The situation will be very harmful if specific action is not taken now to reduce the risk of malaria infection in Benin. Several authors have shown that there is a strong relationship between the prevalence of malaria and climatic variables [8–11]. In Northern Benin, meteorological factors have a strong link with the incidence or prevalence of malaria [12, 13]. Rainfall ensures the persistence of larval sites with continuous vector biting rates in Benin [14].

Modelling the links between climatic factors and the incidence of malaria can help provide a good idea of the relationship between the incidence of malaria and climatic factors. A malaria early warning model can provide insights and indications to researchers and public health decision makers about future outbreak and risks in Northern Benin.

To model the risk of contracting malaria, various statistical techniques were used [9, 10, 15–17]. The majority of approaches [18] are unable to identify the direct and indirect impacts of climatic factors on the incidence or prevalence of malaria. The specification of direct and indirect effects is possible with the structural equation model (SEM) [19]. Latent variables can be included in SEM, such as the idea of "socio health characteristics," which is made up of a number of indicators and is theoretically supported by the confirmatory factor analysis built into SEM [20]. In this study, we used SEM methodology to investigate the causal connections among meteorological variables and their effects on malaria transmission.

## Study site, population, and climate

**Fig. 1:**
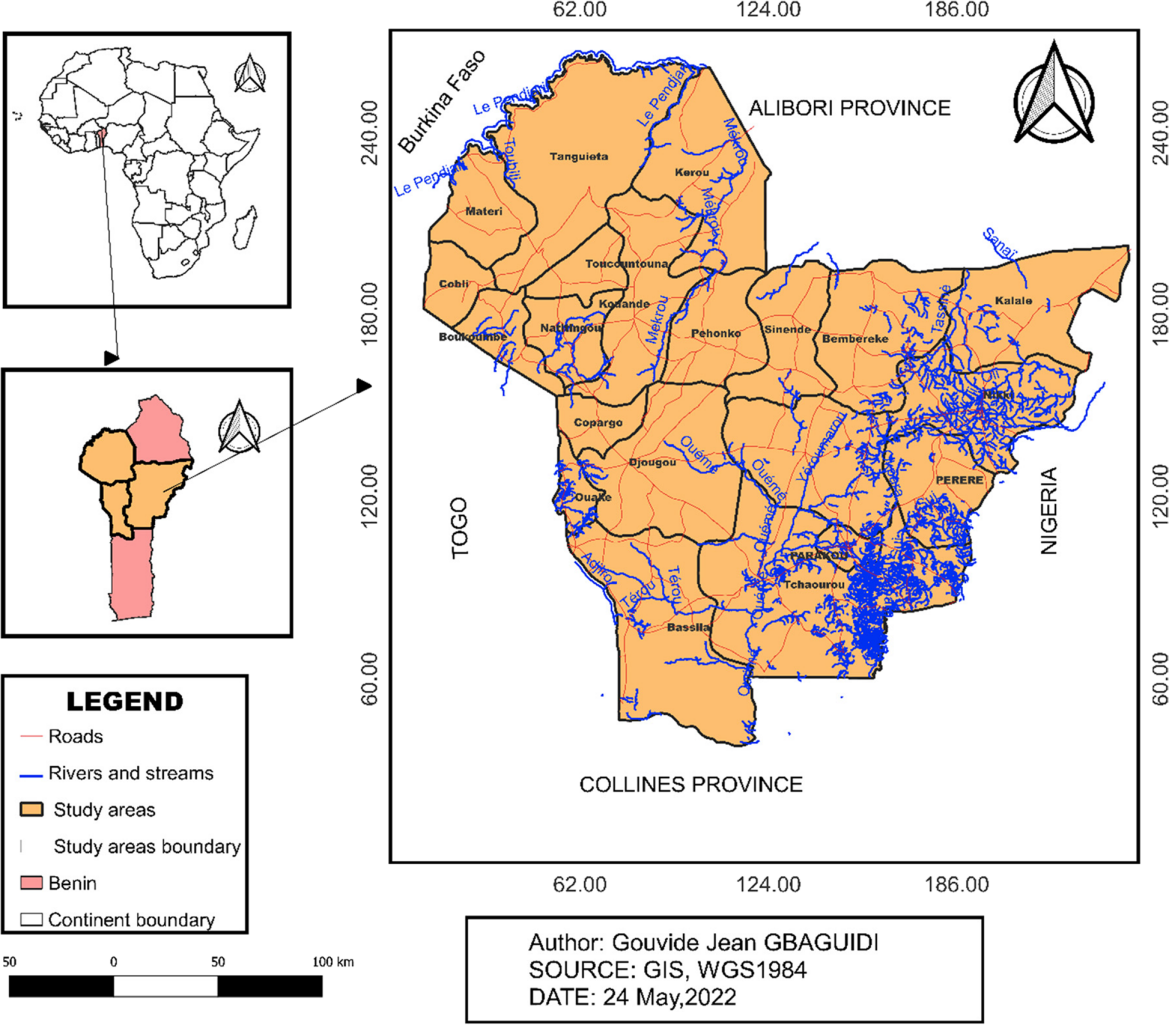
Map of the study area

The study area is located in the northern part of Benin and covers the provinces of Atacora, Donga, and Borgou (Fig. 1). The climate of the study area is hot and humid [21] and is characterized by one dry season and one rainy season. The rainy season lasts from May–October when the ITCZ is in its northern position. Rainfall is maximal (253.61 mm) in August and minimal (1.90) in January. The dry season is from November–April when the ‘Harmattan’ winds blow in from the northeast, bringing air from the Sahara Desert [22].

## Data collection

### Dependent variables

Monthly malaria cases in each district of the provinces of Borgou, Atacora, and Donga were collected from January 2009 to December 2021 at the Ministry of Health of Benin. These data include the entire population confirmed to be infected by malaria. A patient is declared to have malaria when the disease is confirmed at a laboratory by microscopy or rapid diagnosis [23]

### Independent variables

Monthly weather data on temperatures (maximum, mean, and minimum), wind speed, and relative humidity (maximum, mean, and minimum) were retrieved over the period 2009 to 2021 from the meteorological stations of Parakou Airport and Natitingou operated by the National Meteorological Agency of Benin. Monthly precipitation data for the period of 2009 to 2021 from seventeen rain gauge stations were extracted from the rain gauge stations and meteorological stations summarized in Table 1.

**Table 1:** Locations of rain gauges and meteorological stations

Station	Latitude DEG°MIN'SEC''	Longitude DEG.FRAG	Longitude DEG°MIN'SEC''	Latitude DEG.FRAG
BIRNI	09°59′24''	1.529987	001°31′48''	9.990087
PENESSOULOU	09°14′24''	1.551297	001°33′05''	9.239867
TANGUIETA	10°37′02''	1.266561	001°16′00''	10.61712
KOUANDE	10°19′55''	1.692409	001°41′33''	10.33185
NATITINGOU	10°19′00''	1.383333	001°23′00''	10.31667
KALALE	10°17′24''	3.381675	003°22′54''	10.29007
BEMBEREKE	10°12′00''	2.666667	002°40′00''	10.2
BOUKOUMBE	10°10′00''	1.1	001°06′00''	10.16667
NIKKI	09°56′00''	3.2	003°12′00''	9.933333
BASSILA	09°01′22''	1.665544	001°39′56''	9.022698
MATERI	10°43′48''	1.012	001°00′43''	10.73
COPARGO	°'''		°'''	
PEHUNCO	10°13′48''	2.003	002°00′11''	10.2299
PERERE	°'''		°'''	
N'DALI	09°51′00''	2.7	002°42′00''	9.85
PARAKOU_AEROPORT	9°21′00''	2.6	2°36′00''	9.35
NATITINGOU_PEPORIYAKOU	10°22′42''	1.358889	001°21′32''	10.37833

## Data processing

### Factor analysis

The monthly average incidence of malaria and the climatic data were matched. Univariate normality and multivariate (Henze-Zirkler and Anderson‒Darling) tests were used to evaluate the data distribution. The conventional statistical procedure distribution was used to perform analyses of correlation, regression, and collinearity.

### Exploratory Factor Analysis (EFA)

Exploratory factor analysis (EFA) is used to identify confounding latent ecological factors from the collection of observable environmental variables affecting the transmission of malaria [24, 25].

Varimax rotation was employed to determine the connection between the latent components and indicators (Table 2). There were a variety of undiscovered factors influencing malaria infection. Data fitting using correlation and regression analysis, as well as exploratory factor analysis, is used to create the initial model.

**Table 2:** The Varimax method rotated each indicator in the load factor on the two potential factors

Indicators	Factors	Factor1(F1)	Factor2(F2)
I1	Monthly Average Precipitation		
I2	Monthly Average Minimal Temperature		**0.98**
I3	Monthly Average Maximal Temperature	**-0.82**	
I4	Monthly Average Mean Relative Humidity	**0.98**	
I5	Monthly Average Maximal Relative Humidity	**0.94**	
I6	Monthly Average Wind Speed		0.37
I7	Monthly Average Malaria Incidence	0.48	

The model was further scrutinized based on the parameter estimates, the model's applicability of the correlation coefficient of the equation in the model, and the model fitting indicators falling within an acceptable range [26].

### Confirmatory Factor Analysis (CFA)

Confirmatory factor analysis's main objective is to use a single latent variable to shed light on the covariances or correlations between a large number of observed variables [19]. In the prior model, seven observed indicators were examined. After 30 iterations, weighted least squares were employed to assess the convergence of the parameters in compliance with the model t rule (t = p * (p + 1)/2 = 7 * 8/2 = 28) [27].

### Structural Equation Model (SEM)

Structural equation models (SEM) were used to carry out the confirmatory analysis [20]. The distinction between dependent variables and independent variables takes precedence over the distinction between latent and observable variables [25]. Covariances between dependent variables or between dependent variables and independent variables do not vary freely; rather, the model's free parameters are used to describe them. According to [18], the hypothesized model structure placed the correlations between the independent variables at zero.

**Fig. 2:**
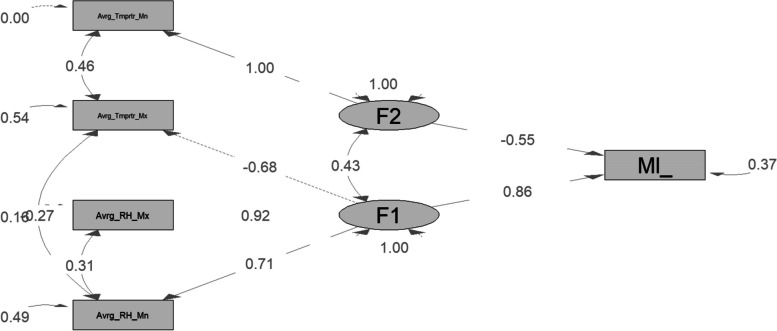
The initial model shows the relationship between malaria incidence and climate factors; the black gray rectangle indicates measurement variables, while the black gray ellipse is the latent variable

The SEM approach is defined by the following system of Eq. (1), where the observed variables can be written as a linear combination of the potential components plus residual terms. Thus, we provide the following mathematical SEM representations of Fig. 2:

1 $$\left\{\begin{array}{l}\mathrm{Factor\ I}= \lambda \mathrm{1,1}(\mathrm{Average\ mean\ relative\ humidity}) + \lambda \mathrm{1,2}(\mathrm{Average\ maximum\ relative\ humidity}) \\ + \beta \mathrm{1,2}(\mathrm{Average\ Maximum\ temperature}) + \gamma 1(\mathrm{malaria\ incidence}) + e1\\ \mathrm{Factor\ II}= \lambda \mathrm{2,1}(\mathrm{Average\ minimum\ temperature}) + \gamma 2(\mathrm{malaria\ incidence}) + e2\end{array}\right.$$

*where λ1,1, λ1,2, λ2,1, β1,2, and γ1, γ2 are coefficients or weights assigned to the respective variables in the equations, representing their relative importance or contribution to the factors.and (e1, e2) are the residual terms.*

### Machine learning

A famous quote attributed to George Box is “All models are wrong; some models are useful” [28] that will be used to make an accurate prediction of malaria incidence. We used a random sampling method to define a training set and test set to train the model and to evaluate the performance of the models to predict. As our number of observations is small, we randomly selected 90% of the data for training and 10% for testing a predictive model. To develop the malaria outbreak warning model, we applied three different machine learning algorithms, including the support vector machine (SVM), linear regression (LiR), and negative binomial regression (NBiR) models.

To find the best prediction algorithm, we calculated the mean absolute error (MAE), mean square error (MSE) and root mean square error (RMSE) of the proposed models (SVM, LiR and BiRM) on the prediction of malaria incidence [29].

## Results

### Exploratory Factor analysis (EFA)

Two latent factors were identified: factor 1 (related to average mean relative humidity, average maximum relative humidity, and average maximal temperature) and factor 2 (related to average minimal temperature). At an *α* = 5% level of significance, *χ*2 = 18.56, df = 8, P value = 0.017, the two identified factors explained 67% of the total variation. This finding offers an adequate explanation for the prevalence of malaria in the study area.

We investigate the Guttman-Kaiser and Cattell scree plots to find the number of components to be retrieved [30, 31]. The exact number of factors is equal to the number of eigenvalues greater than one in the population correlation matrix (Table 3). We calculated the eigenvalues (2.80, 1.12, 0.29, 0.19, -0.04, -0.09, and -0.20) using the correlation matrix. These values showed that there were two factors that affected the incidence of malaria. According to [32], the number of particularly large eigenvalues in the screen plot test is purportedly correlated with the number of factors used in the study.

**Table 3:** Correlation matrix of climatic variables and malaria incidence

Item	Mean	Std.Dev	Average Precipitation	Average minimal temperature	Average maximal temperature	Average mean relative humidity	Average maximal relative humidity	Average Wind Speed	Average Malaria incidence
Average Precipitation	113.96	71.58	1.00						
Average minimal temperature	21.76	1.40	-0.01	1.00					
Average maximal temperature	33.53	2.25	-0.14	0.16	1				
Average mean relative humidity	65.43	13.03	0.12	0.32	-0.76	1			
Average maximal relative humidity	83.38	11.72	0.06	0.39	-0.67	0.96	1		
Average Wind Speed	2.03	0.37	-0.18	0.36	-0.01	0.04	0.05	1	
Average malaria incidence	28.22	6.10	-0.04	-0.18	-0.48	0.42	0.43	-0.2	1

**Fig. 3:**
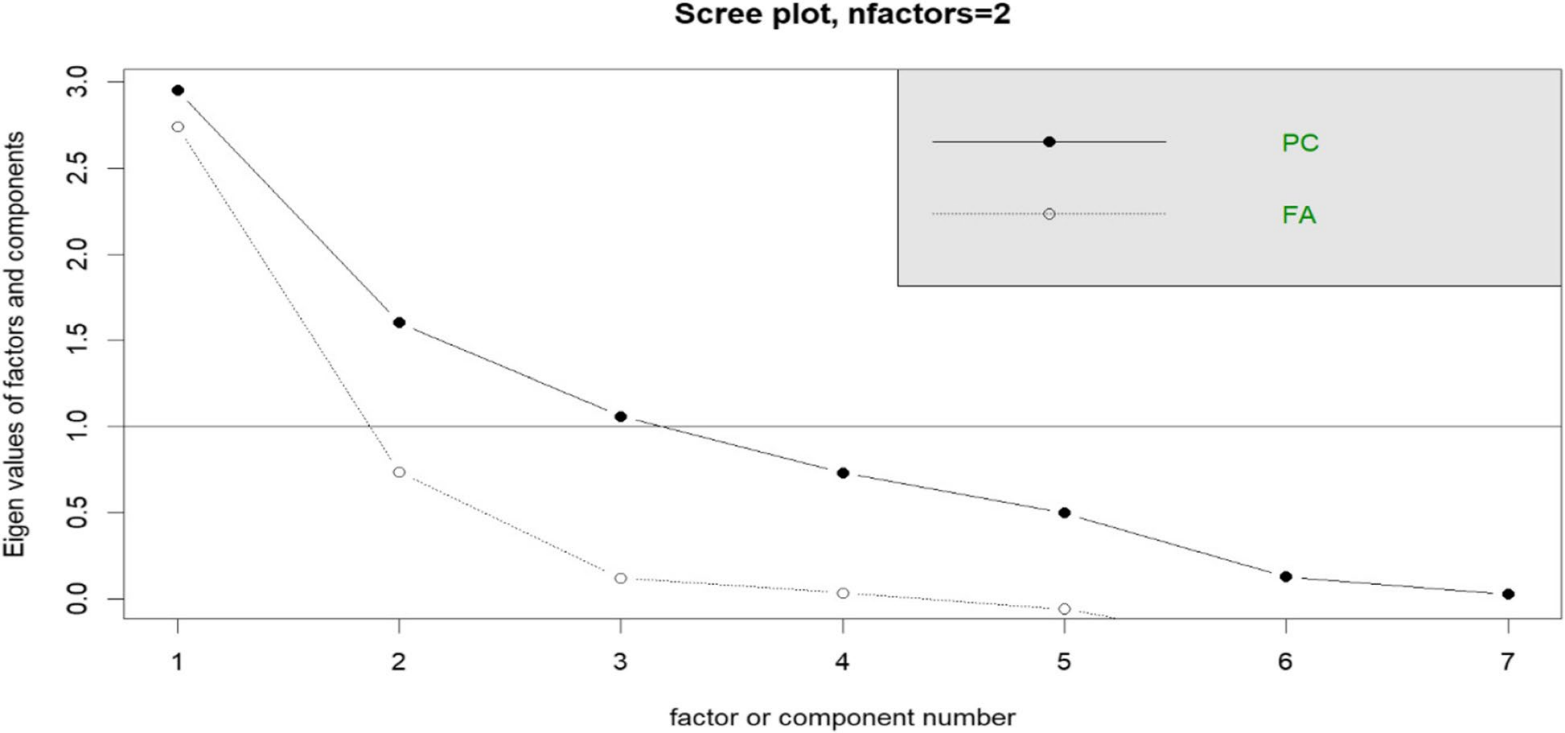
The Cattell scree plot presents the eigenvalues of the components for identifying the number of climatic factors to be considered using the information in Table 3

The scree plot shown in Fig. 3 depicts the relative proportion of variation accounted for by the components and was created from the analysis of the matrix table (Table 3). The parallel indication in the scree plot displays the eigenvalues of the first two components greater than unity, and the succeeding components lower than unity likewise line up beneath the parallel indicator. The scree plot confirmed that there are exactly two latent factors.

Pearson's cross-correlation between ecological variables and the prevalence of malaria at different lag effects from 0 to 3 months is shown in Table 4. The lagged correlation effects between climate variables and the incidence of malaria in Northern Benin are shown by lag0, lag2 and lag3 (e.g., 0 months, 1 month and 2 months) in Table 4. Average precipitation, average mean relative humidity, and average maximum relative humidity all exhibit a positive correlation with the incidence of malaria at lag effects of 0 months and 1 month, with respective values of (0.05,0.423, 0.431) and (0.015,0.554, 0.576). The average mean relative humidity, average maximum relative humidity, and wind speed all had positive correlations with the incidence of malaria at lag effects of two months (0.526, 0.524, and 0.038, respectively).

**Table 4:** Cross-correlation between climatic variables and malaria incidence

Variables	0 Month	1Month	2 Months
Average Precipitation	0.05	0.015	-0.073
Average minimal temperature	-0.183	-0.007	0.156
Average maximal temperature	-0.483	-0.456	-0.073
Average mean relative humidity	0.423	0.554	0.526
Average maximal relative humidity	0.431	0.576	0.554
Average Wind Speed	-0.195	-0.119	0.038

These findings suggest that meteorological factors at lags of 1 and 2 months would be favourable for mosquito reproduction and the end of their incubation periods (EIPs), which are essential for mosquitoes to transmit malaria vectors to people. Owing to the positive correlation between wind speed and malaria incidence in this study area, the transmission of malaria will be particularly high at a lag effect of two months, and the neighboring districts that are asymptomatic may contract the disease. For the development of mosquito breeding sites and their ability to infect humans, the 1-month lag effects of precipitation and relative humidity are more than sufficient. On the other hand, average minimum temperatures, average maximum and mean relative humidity and average wind speed are all excellent for mosquito development and disease transmission on a high scale.

### Confirmatory factor analysis

**Fig. 4:**
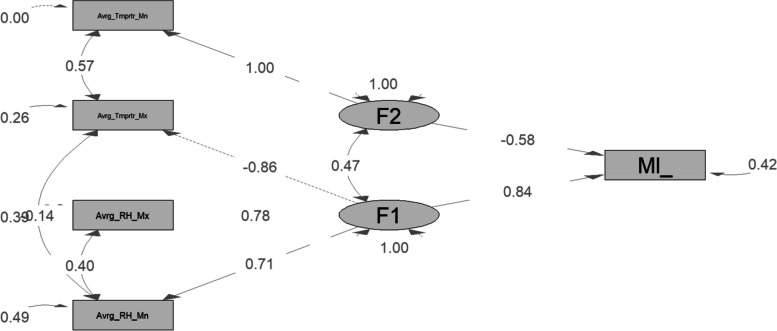
The final model path diagram shows the relationship between malaria incidence and climate factors; the black gray rectangle indicates measurement variables, while the gray ellipse is the latent variable

CFA was used to confirm how many factors should be extracted from the meteorological variables. Analysis was performed on seven observed indicators. Four observable indicators were kept in the final model thanks to two latent components. Upon the initial model's correction (Fig. 4), the fit indices were significantly better than they had been, and the standardized residual distribution was smaller than it had been with the initial models. The recommended cut-off values for TLI and CFI are 0.90, and those for RMSEA are 0.06 to validate the CFA model. CFI > 0.90 or RMSEA 0.06 denotes a solid model [33] The robust Tucker‒Lewis index (TLI), robust comparative fit index (CFI), and approximate root mean square error (RMSEA) fitting indices were in the acceptable range, which indicates that the model fits the data very well (TLI = 1, CFI = 1, SRMR = 0.008, and RMSEA = 0). Every latent variable was significant and had a loading above 50%.

### Structural equation model (SEM)

The Henze-Zirkler test revealed the nonnormality of the data set (P value = 0). The means, standard deviations, and bivariate correlations for all variables included in the analysis are shown in the correlation matrix Table 3. Multicollinearity among the variables was discovered through study of the correlation matrix table. Numerous predictors are highly correlated, notably among the meteorological factors, as seen by the size of the connections. Given the SEM analytical technique's superior handling of intercorrelated independent variables through the development of latent constructs and direct and indirect pathways, which avoid the propensity to distort coefficient estimates, this is one of the requirements for using it [20].

A graphic depiction of the model under analysis is shown in Fig. 2. It is vital that suggested cut-off values are confidently within an acceptable range before looking at the relationships shown in the model. When the model shown in Fig. 2 is examined, the following values are found: chi-square (348.113 df = 10, not significant), RMSEA (0.000), TLI (1), CFI (1), and SRMR (0.008). These numbers demonstrate that the model successfully fits the data and may be applied to determine how ecological factors affect the prevalence of malaria.

### Effects of climatic variables on the incidence of malaria

The analysis of the model (Fig. 2) reveals that the direct effect of factor 1 is 0.84 and that of factor 2 is -0.58. The direct effects of average maximal temperature, average maximal relative humidity, average mean relative humidity, and average minimum temperature on the incidence of malaria were -0.86, 0.78, 0.71 and 1, respectively. The indirect effects of average mean relative humidity, average maximal temperature and factor 2 are indicated as 1.59, 1, and 0.39, respectively.

0.78, 2.30, 0.14, 1, 0.84, and -0.19 are the total respective effects of average maximal relative humidity, average mean relative humidity, average maximal temperature, average minimal temperature, factor 1 (F1), and factor 2 (F2). Among the two factors identified by EFA, factor 1 had the highest direct effect and the highest total effect on the incidence of malaria in the study area. We conclude that factor 1 is the most influential hidden climatic factor in the incidence of malaria. Therefore, average factor 1 can be used to model and predict the incidence of malaria in the northern part of Benin.

### Intelligent malaria outbreak warning model

The next stage is to choose the algorithm that would accurately forecast the prevalence of malaria in the study areas. The most significant hidden climatic element in the prevalence of malaria was discovered in the preceding section as factor 1, which was indicated by average maximal relative humidity, average mean relative humidity, and average maximal temperature.

**Fig. 5:**
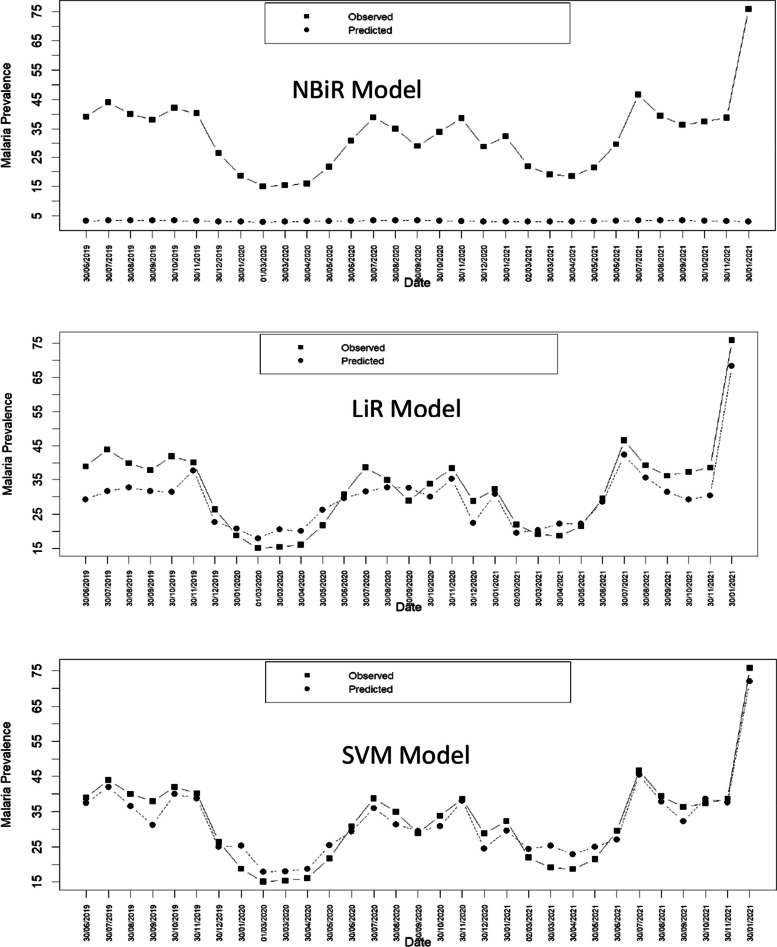
Test of the incidence of malaria using SVM, NBiR and LiR

To develop the malaria outbreak warning model, we applied three different machine learning algorithms, including support vector machine (SVM), linear regression (LiR), and negative binomial regression (BiR) models. After training and testing the different algorithms (Fig. 5), we assessed the performance of each model to identify the best algorithm that has good accuracy in predicting the incidence of malaria in the northern part of Benin (Table 5).

**Table 5:** Assessment of the Model Performance

Item	MAE	MSE	RMSE	Predicted R2
LiR	2.50	11.07	3.33	66%
SVM	1.66	5.89	2.43	82%
NBiR	22.11	504.84	47.22	66%

Table 5 shows the MAE, MSE, and RMSE for the SVM, LiRM, and BiRM models. The examination of these errors enables us to find that the SVM model offers the best-optimized solution for forecasting more than the two other models. We conclude that the support vector machine (SVM) performs best at predicting the prevalence of malaria in Northern Benin.

### Prediction of malaria incidence under scenarios RCP4.5 and RCP8.5

The RCA4 regional climate model used in this study was developed at the Swedish Meteorological and Hydrological Institute and has provided nearly 120 simulations in the CORDEX project (Coordinated Regional Climate Downscaling Experiment). The model considers the physical, chemical, and biological processes by which ecosystems affect climate at various spatial and temporal scales. Projected rainfall, temperature and relative humidity were retrieved from the coordinated regional downscaling experiment (CORDEX) simulations of the Rossby Centre Regional Atmospheric regional climate model (RCA4). The CORDEX-Africa data used in this work were obtained from the Earth System Grid Federation server (https://esgf-data.dkrz.de/search/cordex-dkrz/) driven by the RCA4 model. We have predicted the incidence of malaria with the Intelligent Malaria Outbreak Model we built (SVM model) by using the downloaded CORDEX data under two representative concentration pathway (RCP) scenarios (RCP4.5 and RCP8.5) in the northern part of Benin with the RCA4-downscaled driving by the regional climate models RCA4/HadGEM, RCA4/CSIRO, and RCA4/MIROC over the 2021–2030, 2031–2041 and 2041–2050 periods.

**Fig. 6:**
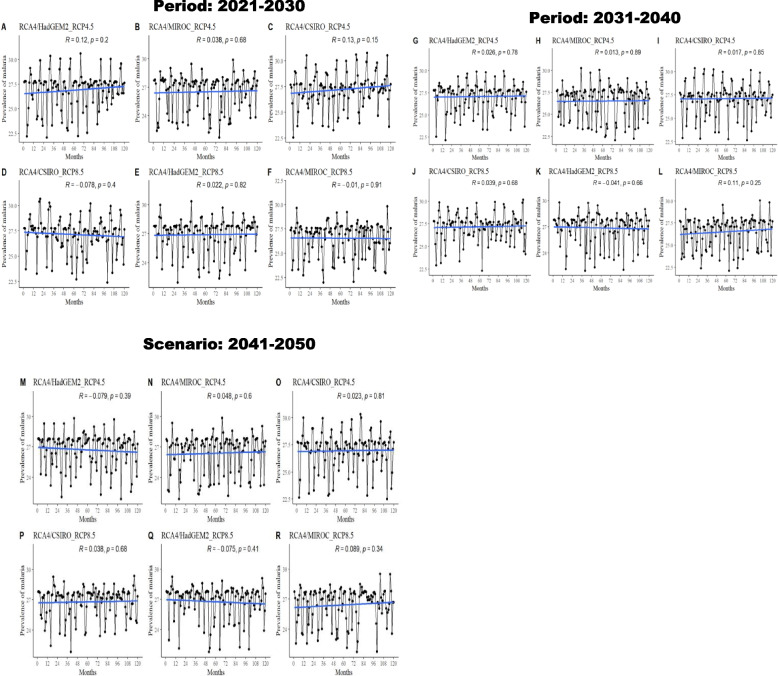
prediction of malaria incidence under RCP4.5 and RCP8.5 by RCA4/HadGEM2, RCA4/CSIRO, and RCA4/MIRO over 2021–2030, 2031–2040 and 2041–2050

Regional climate models RCA4/HadGEM2, RCA4/CSIRO, and RCA4/MIROC over the 2021–2030, 2031–2040 and 2041–2050 periods are associated with an increase in malaria incidence under the RCP4.5 scenario (Fig. 6). RCA4/HadGEM2 is associated with a decrease in the incidence of malaria over the period 2041–2050 under the same scenario.

The incidence of malaria will decrease under the RCP8.5 scenario driven by RCA4/CSIRO and RCA4/MIROC over the 2021–2030 period (Fig. 6). An increase in malaria incidence is associated with RCA4/HadGEM2 over the same period under RCP8.5 (Fig. 6).

Overall, the findings suggest that malaria incidence will increase over 2021–2050 under scenarios RCP4.5 and RCP8.5 except for the 2021–2030 period, when the incidence of malaria will decrease under RCP8.5 due to climate change. With regard to the findings of this study, the northern part of Benin is at high risk of malaria.

## Discussion

The malaria early warning model can be a good tool in the control of malaria transmission. In the northern part of Benin, climatic factors indicated by average mean relative humidity, average maximum relative humidity, and average maximal temperature are the most influential meteorological variables of malaria infection. This factor has a positive effect on the incidence of malaria. This result is consistent with the finding of [27], where malaria incidence is positively associated with minimum temperature and relative humidity.

The projection revealed that malaria incidence will increase over 2021–2050 under scenarios RCP4.5 and RCP8.5, except for the 2021–2030 period, when the incidence of malaria will decrease under RCP8.5 due to climate change. An increase in malaria incidence will be observed in the northern part of Benin over the 2021–2030, 2031–2040 and 2041–2050 periods under the RCP4.5 scenario. Climate change is increasing the risk of malaria infection in the study areas. A study carried out in Northern Benin found that climate change has a real impact on Anopheles’ density and weakens current and future vector control strategies [34]

Climate changes or different weather conditions may impact infectious diseases, specifically, those transmitted by insect vectors and contaminated water [6, 35–37]. Afrane et al. [38] confirmed that temperature increases promote the rapid digestion of blood supply, which in turn promotes a significant increase in fecundity, with the development of better reproductive fitness and a greater ability to produce more offspring.

A study conducted by Salako in the province of Alibori, Northern Benin, confirmed that the biting rates of An. Gambiae is higher in the rainy season than in the dry season [13]. Subtil et al. [14] study in South Benin certified that rainfall ensures the persistence of peri-domestic larval sites in villages with continuous vector biting rates. The risk of malaria infection is very high in the rainy season in the northern part of Benin. These findings confirmed the influence of climate change on the transmission of malaria in the northern part of Benin.

The study carried out by [17] in West Africa found that the prevalence of malaria is expected to increase in the southern part of the region in the future. This result was consistent with our study, which revealed an increase in the prevalence of malaria under RCP4.5 and RCP8.5.

The use of climatic factors to predict the risk of malaria infection also agrees with the findings of [39], who developed a simple model of climate-related malaria transmission that provides insights into the sensitivity of disease transmission to changes in precipitation and temperature. The consideration of climate variables in surveillance systems, as well as the integration of future climate projections into epidemiological models to more effectively predict the prevalence and outbreaks within the context of a changing climate, is very important [4, 40]

The increase in malaria incidence over the 2021–2050 period is supported by the projection of the IPCC that predicted that malaria may threaten some previously unexposed regions of South America and sub-Saharan Africa (SSA) in 2050 under the current climate change associated with the increase in CO2 concentrations and increase in atmospheric temperature [2].

The influence of urbanisation on malaria risk in African cities is significant [41]. While Anopheles mosquitoes have been discovered to adapt to urban development sites over time, they are known to breed more in rural settings [42]. Anopheles mosquito resistance to antimalarial drugs in Africa hinders malaria efforts due to environmental factors [43], despite attention being directed towards addressing this issue.Continuous monitoring and evaluation of current and future malaria transmission status in Northern Benin is a mainstay for the success of ongoing intervention strategies for malaria control.

## Conclusion

In light of the work above, climate factors determine the transmission of malaria in Northern Benin. Relative humidity and temperature have a potential influence on the transmission of malaria in the study areas. Relative humidity and maximal temperature favor the development of mosquitoes breeding sites and increase the transmission of malaria.in Nord Benin. An intelligent malaria outbreak warning model developed by employing a support vector machine predicts the incidence of malaria at 82% in the study areas. This study reveals an increase in malaria incidence over the 2021–2050 period under two different scenarios, RCP4.5 and RCP8.5. The incidence of malaria will decrease under the RCP8.5 scenario over the 2021–2030 period. The findings of this study are a powerful tool for stakeholders to take specific preparedness actions to lessen the impacts of climate change on human health by reducing the emission of greenhouse gases in the study area.

## Data Availability

Data are available at the West African Science Service Centre on Climate Change and Adapted Land Use (WASCAL), Université de Lomé. You should contact the corresponding author for the data request.
